# Educational intervention with nursing professionals reduces interruption of enteral nutritional support*

**DOI:** 10.1590/1980-220X-REEUSP-2024-0132en

**Published:** 2024-09-09

**Authors:** Aline Oliveira Diniz, Igor Rosa Meurer, Kely Cristine Batista, Valesca Nunes dos Reis, Ana Paula Boroni Moreira, Silvia Lanziotti Azevedo da Silva

**Affiliations:** 1 Universidade Federal de Juiz de Fora, Hospital Universitário, Empresa Brasileira de Serviços Hospitalares, Juiz de Fora, MG, Brazil.; 2 Universidade Federal de Juiz de Fora, Hospital Universitário, Juiz de Fora, MG, Brazil.; 3 Universidade Federal de Juiz de Fora, Instituto de Ciências Biológicas, Departamento de Nutrição, Juiz de Fora, MG, Brazil.; 4 Universidade Federal de Juiz de Fora, Faculdade de Medicina, Departamento de Saúde Coletiva, Juiz de Fora, MG, Brazil.

**Keywords:** Enteral Nutrition, Energy Intake, Early Intervention, Educational, Nursing, Nutrición Enteral, Ingestión de Energía, Intervención Educativa Precoz, Enfermería

## Abstract

**Objective:**

To evaluate the impact of educational intervention on the occurrence of factors that interfere with the caloric-protein supply to critical and non-critical patients undergoing enteral nutritional therapy.

**Method:**

This is an intervention, a field experiment without a control group, carried out in a teaching hospital in Juiz de Fora, Minas Gerais, Brazil. Three training cycles were carried out with nursing professionals over 57 weeks, covering the same content. The data collected were divided into pre-intervention and intervention periods. Interference in caloric-protein support was analyzed based on the evaluation of days with non-conforming nutritional supply and the occurrence of factors that led to non-conformities.

**Results:**

Following interventions, there was a significant reduction (p < 0.05) in the number of non-conforming and inadequate days (nutritional supply < 80%), the frequency of occurrence of interfering factors and non-conformities caused by failure to follow the vomiting protocol.

**Conclusion:**

The educational intervention was an effective strategy to increase the quality of the therapy evaluated, especially from the fifth day of enteral nutritional therapy onwards.

## RESUMEN

**Objetivo:**

Evaluar el impacto de la intervención educativa en la aparición de factores que interfieren en el aporte calórico-proteico de pacientes críticos y no críticos sometidos a terapia nutricional enteral.

**Método:**

Se trata de una intervención, un experimento de campo sin grupo de control, realizado en un hospital universitario en Juiz de Fora, Minas Gerais, Brasil. Se realizaron tres ciclos formativos con profesionales de enfermería durante 57 semanas, abarcando los mismos contenidos. Los datos recopilados se dividieron en períodos de preintervención e intervención. Se analizó la interferencia en el aporte calórico-proteico a partir de la evaluación de los días con incumplimiento de la provisión nutricional y la ocurrencia de factores que llevaron a las no conformidades.

**Resultados:**

Después de las intervenciones hubo una reducción significativa (p < 0,05) del número de días de incumplimiento e inadecuados (suministro nutricional < 80%), la frecuencia de aparición de factores de interferencia y no conformidades provocadas por la no ejecución del protocolo de vómitos.

**Conclusión:**

La intervención educativa resultó una estrategia efectiva para incrementar la calidad de la terapia evaluada, especialmente a partir del quinto día de terapia nutricional enteral.

## INTRODUCTION

The association between Enteral Nutritional Therapy (ENT) and the patient’s clinical progression is already very well established, as it is a therapeutic strategy capable of reducing disease severity, infectious complications, length of hospital stay and mortality^(1,2)^. Such benefits are conditioned on its early implementation (first 48 hours), meticulous glycemic control, and adequate supply of calories, macro and micronutrients, with ENT being the preferred route of administration, whenever feasible^(2)^.

Despite the benefits, hospitalized patients commonly do not receive the sufficient prescribed nutritional support to meet their demands^(3)^. Some studies evaluated the provision of enteral diets in different patient profiles and found inadequacies in relation to caloric and protein prescriptions: two studies conducted in intensive care units found that 30%^(4)^ and 25%^(5)^ of patients did not reach the caloric-protein goal; research carried out in an oncology hospital^(6)^ found that only 50.2% of the prescribed diet volume was received; and in another study carried out with critically ill patients^(7)^ the authors found that, although 92% of patients received more than 80% of the programmed volume, only 43.6% received more than 80% of the protein target, with an association being found between protein inadequacy and mortality.

The occurrence of nutritional deficits is due to several conditions that interfere with the planned nutritional supply, leading to its temporary and/or permanent suspension, such as gastrointestinal symptoms (vomiting, diarrhea, high gastric drainage, abdominal distension, constipation), externalization/obstruction of the feeding catheter, fasting for procedures and exams, and hemodynamic instability^(6,8,9)^. Inadequate practices by professionals in the multidisciplinary team, such as inadequate prescription, late start of ENT and unnecessary pauses, as well as patient refusal of treatment, are still cited as factors leading to interruption of the diet^(4,10)^.

Knowledge of the factors that impair satisfactory energy-protein administration allows the adoption of measures to minimize interruptions in the diet and ensure adequate administration of ENT. Examples of these factors include nutritional protocols improvement, establishment of control measures for gastrointestinal complications, evaluation of the duration of interruption for exams and procedures, training of teams to better apply protocols and better record information^(5,9,11)^.

Nutrition has been an important field of action for nursing since the 19th century, when the founder of modern nursing already carried out actions in favor of adequate and good quality nutrition^(12)^. Nursing care is vital to the success of ENT. These include monitoring of the patient and the potential complications, maintenance of catheter functionality, performance of oral and nasal hygiene, reduction of the risk of aspiration, and adequate recording of the solution volumes administered^(13,14)^.

The literature available highlights the need to review practices and constant nursing training in health services, especially those involved with the care of patients using ENT, with the expansion of practical knowledge on this topic^(12,15)^. Considering that the occurrence of several factors hinders the achievement of nutritional goals and, also, the gaps in the training of professionals in the care team, the need to increase the effectiveness of ENT to ensure its quality and benefits for the patient’s progression is evident.

Therefore, the present study aims to evaluate whether a dialogic educational intervention aimed at nursing professionals involved in the administration of ENT is capable of minimizing the occurrence of factors that interfere with enteral nutritional provision to critical and non-critical patients admitted to a university hospital.

## METHOD

### Design of Study

Field experiment study without control group.

### Local

The research was carried out at the University Hospital of the Universidade Federal de Juiz de Fora, a city in the state of Minas Gerais, Brazil.

### Target Population of the Experiment

The experiment was aimed at nursing professionals who work to assist adult and elderly patients admitted to the Hospital.

### Educational Strategy: Carrying Out Training

**Figure 1:**
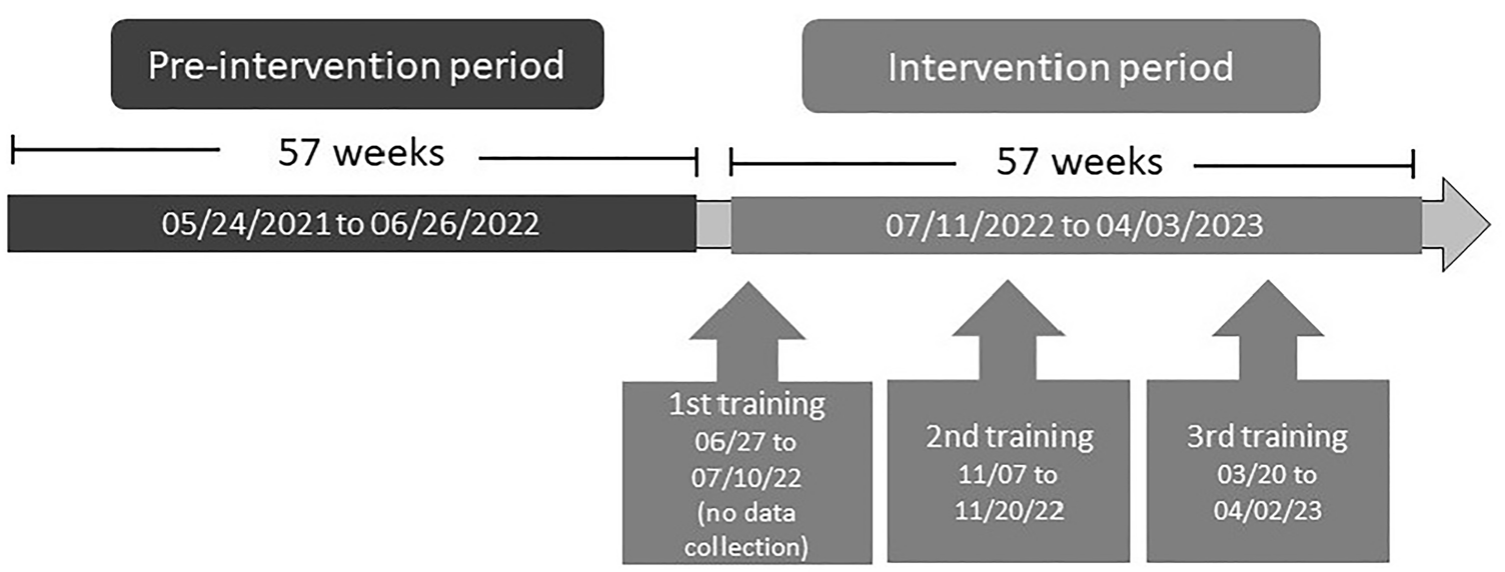
Flowchart of the pre-intervention and intervention periods.

The study was conducted between May 2021 and August 2023, being divided into two periods: pre-intervention period (Ppi) and intervention period (Pi). Figure 1 illustrates the study design and periods.

The educational intervention consisted of three training cycles developed throughout the intervention period (Pi), with 19-week intervals between them.

Aiming to reach a greater number of nurses and nursing technicians, the training was previously scheduled with the coordinators of each sector (wards and Intensive Care Unit - ICU), taking place in three work shifts (morning, afternoon, and night), on strategic days, during working hours and at the employees’ respective workstations, with an average duration of 30 minutes. Training was carried out for each of the 17 nursing teams participating in the study in each of the 3 cycles, totaling 51 trainings at the end of the study.

Considering that the nursing professionals worked in different shifts (morning and/or afternoon or night) and that some were possibly on vacation, time off, or away during some of the intervention moments, the trained participants were encouraged to be multipliers of the content covered.

The training was provided by two nurses from the Multidisciplinary Nutritional Therapy Team (*EMTN*) with the support of a nutritionist from that team, using the Talking Circles strategy. Therefore, they were conducted in such a way that they consisted of meeting spaces with workers from different professional categories with opportunities for dialogue and reflection on the factors that imply the inadequate administration of the ENT, seeking the best behavior for their conduction^(16)^.

The basic content covered in the training was prepared according to the Nursing Guidelines in Nutritional Therapy of the Brazilian Society of Parenteral and Enteral Nutrition (BRASPEN)^(14)^ and the Collegiate Board Resolution - RDC nº 503^(13)^, which provides for the minimum requirements for ENT, consisting of the following topics: a) Maintenance of patency, unblocking techniques, and control of the positioning of catheters for ENT; b) Compliance with the institutional vomiting protocol; c) Occurrence of diarrhea; d) Recording of information; e) Management of the controlled infusion pump (CIP); f) Instructions regarding the execution of the nutritional prescription.

The same contents were covered in the three training cycles, favoring better knowledge retention and standardization of the methodology used.

### Data Collection

ENT patients admitted to the studied institution are daily monitored by the *EMTN*. Calculations of caloric and protein requirements are carried out in accordance with institutional protocols and follow the recommendations of the NT Guidelines of institutions such as the American Society for Parenteral and Enteral Nutrition (ASPEN)^(1)^, ESPEN (European Society for Clinical Nutrition and Metabolism)^(2)^ and Braspen (Brazilian Society of Parenteral and Enteral Nutrition)^(17)^.

The evaluation of the effectiveness of this intervention on the administration of ENT was performed through the analysis of variables related to the days of interruption of the diet with or without inadequate supply of calories and proteins and the occurrence of factors that interfere in this supply, with the same variables being compared in the two periods considered. This way, information regarding the prescription and infusion of ENT and factors that interfered in the provision of ENT to adult and elderly patients (19 years or older) of both sexes, admitted to the ICU and hospital wards and who were on ENT via an exclusive catheter were included. Information from patients who were on ENT for less than 72 hours, were pregnant, and were undergoing palliative care/terminal illness was excluded.

Ppi data were collected before the start of the first training, for the same time as Pi (57 weeks). To evaluate the impact of the intervention on nutritional supply, ENT days were evaluated according to whether or not the caloric-protein supply complied with the prescribed values, with those days when there was any interference in nutritional support that led to a ­caloric-protein supply different from the one prescribed being considered non-compliant days. Likewise, based on the evaluation of non-compliant days, the days on which the caloric-protein supply was equal to or less than 80% of the prescribed amount were quantified, as it has already been demonstrated that the ideal caloric load associated with better survival is around 80% of predicted energy requirements^(1,2)^. The occurrence or not of factors that interfered with the provision of ENT in each service provided by the teams was also analyzed: at medical discretion, complications related to the gastrointestinal tract (GIT), catheter obstruction, externalization of the catheter, clinical complications, fasting, failure to comply with the prescription, failure to follow the ENT vomiting protocol, others, and undetermined.

To achieve the clinical benefits of ENT during the first week of hospitalization, more than 80% of the estimated caloric and protein goals must be provided within 48 to 72 hours after starting nutritional support and full nutritional support must be prescribed from 3 to 7 days from the start of EN^(1,2)^. Therefore, the progression of the infusion speed of enteral diets follows the protocol established by the *EMTN*, starting with 50% (first day of ENT – D1) and progressing to every 24 hours, after team assessment, to 65%, 80% and 100% of calculated needs. Thus, the dietary prescription achieves 100% adequacy to caloric and protein needs on the fifth day (D5) of nutritional support. Thus, seeking to better elucidate on which days of ENT the support provided to patients is most affected (critical point), the services were subdivided according to the days of ENT: services carried out from the first (D1) to the fourth day (D4) of ENT and care provided from the fifth day onwards (D5).

The data collected refers to the care provided by the Nutrition and Nursing teams, whose standard is independent of the profile of the patient to whom they are directed as they are determined by institutional protocols.

### Statistical Analysis

Mean and standard deviation values for the number of ­non-conforming days were calculated, specifying those days with adequate (>80%) and inadequate (≤80%) supply, and the number of occurrences of factors interfering with the provision of ENT in Ppi and Pi. The absolute and relative frequencies of occurrence of each of these factors in the care provided by the teams in the two collection periods were also calculated.

To evaluate the effectiveness of the intervention, the same variables were considered in Ppi and Pi. The association between the two evaluation periods and the possible effectiveness of the intervention was verified using mixed linear regression models. According to the values of the Beta coefficient in Pi, compared to Ppi, it was possible to identify whether there was an average increase or decrease in each variable in Pi, both in the first four days of ENT (D1 to D4) and from the fifth day onwards (D5+). Mixed linear models allow analyzing the fixed effect of the intervention carried out, controlling for the random effect resulting from the profile of patients treated in each period. Random effect control in the mixed linear model minimizes possible effects of patients’ individual characteristics, which were not the same in all intervention periods. The fixed effect, the one that is actually evaluated, is the effectiveness of the educational intervention on the care provided, which is carried out by the same professionals, the target population of the intervention, in all periods.

To compare the interruption frequencies for each of the factors studied between Ppi and Pi, the McNemar test was used to compare proportions. The test was chosen based on the dependency between the moments, considering that the interventions were applied by the same teams.

For the statistical interpretation of the results, the 95% confidence interval (95%CI) was considered and in all tests an alpha significance level lower than 0.05 was adopted. The analyses were performed using the R 4.3.1 statistical package.

### Ethical Aspects

The Human Research Ethics Committee of the University Hospital of the Universidade Federal de Juiz de Fora approved the research through technical opinion number 4.825.877. The professionals who agreed to participate in this study provided their consent by signing the Free and Informed Consent Form.

## RESULTS

Fifty-one training sessions were carried out during the intervention period (Pi), with the participation of 58 nurses and 202 nursing technicians, totaling 260 trained professionals.

There was a gradual increase in the participation of professionals throughout the three training cycles (C1, C2 and C3): 55.33% of Nurses and Nursing Technicians participated in C1, 61.48% participated in C2, and the greatest achievement occurred in C3, when 64.75% of professionals participated.

Among the 58 nurses who participated in the training: 18 (31%) participated in one training, 22 (38%) in two training courses, and 18 (31%) in three training courses. As for the 202 nursing technicians: 106 (52.5%), 67 (33.2%) and 29 (14.3%) participated, respectively, in one, two, or three training courses.

Considering that the training also had the purpose of promoting communication and integration between the Nutrition and Nursing care teams, on some occasions, depending on the subjects’ participation, training duration exceeded the expected, as some teams interacted more than others.

In Ppi, 367 attendances were evaluated, 160 attendances from D1 to D4 and 207 in D5+; and in Pi, 333 attendances were evaluated, 157 from D1 to D4 and 176 in D5+.

In Table 1, the attendances were evaluated according to the number of non-conforming days with inadequate (≤80% of prescribed goals) and adequate (>80% of prescribed goals) caloric-protein supply.

**Table 1:** Average (±SD) of non-conforming days in care evaluated in the pre-intervention (Ppi) and intervention (Pi) periods – Juiz de Fora, MG, Brazil, 2022-2023.

Variable	ENT days	Ppi			Pi		
		n	%*	Mean ± SD	n	%**	Mean ± SD
Total non-conforming days	D1 to D4 and D5+	1220	29.93	5.35 ± 4.50	895	29.12	4.48 ± 4.14
	D1 to D4	255	39.84	1.59 ± 15.81	239	38.06	1.52 ± 1.10
	D5+	965	28.08	4.66 ± 0.95	656	26.82	3.73 ± 4.06
Non-conforming and inadequate days (≤ 80%)	D1 to D4	114	17.81	0.71 ± 1.19	81	12.90	0.52 ± 0.71
	D5+	533	15.51	2.57 ± 4.40	354	14.47	2.01 ± 2.51
Non-conforming and adequate days (> 80%)	D1 to D4	141	22.03	0.88 ± 0.90	158	25.16	1.01 ± 0.96
	D5+	432	12.57	2.09 ± 2.57	302	12.35	1.72 ± 2.10

*In relation to the 4076 days evaluated, 640 days from D1 to D4 and 3436 on D5+.

**In relation to the 3074 days evaluated, 628 days from D1 to D4 and 2446 on D5+. ENT – Enteral Nutritional Therapy, Ppi – Pre-intervention period, Pi – Intervention period.

It could be stated that the supply of calories and proteins was in compliance with the dietary prescription on the majority of ENT days in the two periods evaluated: in 2856 (70.07%) and 2179 (70.88%) days evaluated in Ppi and Pi, respectively, the caloric-protein supply was similar to the prescribed values. In its turn, caloric-protein inadequacy (supply ≤ 80% of goals) occurred in 647 days (15.87%) in Ppi and 435 days (14.15%) in Pi. It is also noted that in Pi there was a reduction in the mean values for all variables analyzed, except for the non-conforming and adequate days evaluated in the first 4 days of ENT (Table 1).

Table 2 presents the average number of factors, considering all those studied together, that interfered with the provision of ENT in the evaluated services. It is possible to see that the factors interfered more in the care provided from the fifth day of ENT and, also, that there was a drop in the average number of factors in Pi.

**Table 2:** Mean (±SD) frequency of occurrence of factors that interfered with the provision of ENT in care evaluated in the pre-intervention (Ppi) and intervention (Pi) periods – Juiz de Fora, MG, Brazil, 2022-2023.

Variable	ENT days	Ppi		Pi	
		n	Mean ± SD	n	Mean ± SD
Number of interference factors	D1 to D4	307	1.92 ± 2.56	286	1.82 ± 1.49
	D5+	1108	5.33 ± 5.39	727	4.13 ± 4.77

ENT – Enteral Nutritional Therapy, Ppi – Pre-intervention period, Pi – Intervention period.

As shown in Table 3, the Beta values showed a reduction in the average number of non-conforming and inadequate days in Pi, both in the first days of ENT (D1 to D4) and in subsequent days (D5+), with a statistically significant difference. Furthermore, Beta values demonstrated a reduction in the average number of factors that interfered with nutritional supply from D5 of ENT on Pi, with statistical significance (p = 0.02).

**Table 3:** Mixed Linear Model for non-compliant days and occurrence of factors that interfered in the ENT – Juiz de Fora, MG, Brazil, 2022-2023.

Variable	ENT days	Random effect individual	Fixed effect			
			Intercept	Beta 1	SE	p value
Total non-conforming days	D1 to D4 and D5+	4.85	17.68	–2.63	1.38	0.06
	D1 to D4	0.00	0.00	0.00	0.00	1
	D5+	5.12	16.31	–4.40	1.41	0.00
Non-conforming and inadequate days (=80%)	D1 to D4	0.05	0.71	–0.19	0.09	0.03
	D5+	0.75	2.55	–0.55	0.25	0.02
Non-conforming and adequate days (>80%)	D1 to D4	0.12	0.88	0.12	0.10	0.24
	D5+	0.80	2.04	–0.36	0.22	0.11
Number of interference factors	D1 to D4	0.00	1.91	–0.09	0.18	0.59
	D5+	1.55	5.27	–1.16	0.50	0.02

ENT – Enteral Nutritional Therapy, SE – Standard error.

Table 4 describes the absolute and relative frequencies of each interruption factor in care, considering the total number of services evaluated in each period. It is worth highlighting that the same service can be affected, simultaneously or not, by more than one interfering factor. The table also presents the comparison of these frequencies between Ppi and Pi using the McNamar test.

**Table 4:** Occurrence of factors that interfered with ENT in care provided in the pre-intervention (Ppi) and intervention (Pi) periods – Juiz de Fora, MG, Brazil, 2022-2023.

Interference factors	ENT days	Attendances that suffered interference		McNamar	p value
		Ppi*	Pi**		
At medical discretion	D1 to D4	15 (9.4%)	9 (5.7%)	0.01	1.00
	D5+	33 (15.9%)	25 (14.2%)	0.25	0.61
GIT complications	D1 to D4	14 (8.8%)	15 (9.5%)	0.01	1.00
	D5+	65 (31.4%)	42 (23.9%)	1.04	0.30
Catheter obstruction	D1 to D4	5 (3.1%)	3 (1.9%)	0.8	0.37
	D5+	33 (15.9%)	14 (7.9%)	3.55	0.06
Externalization of the catheter	D1 to D4	21 (13.1%)	21 (13.4%)	0.01	1.00
	D5+	63 (30.4%)	55 (31.2%)	0.40	0.52
Clinical complication	D1 to D4	8 (5.0%)	10 (6.4%)	0.01	1.00
	D5+	51 (24.6%)	31 (17.6%)	1.76	0.18
Fasting	D1 to D4	38 (23.7%)	37 (23.6%)	0.37	0.54
	D5+	121 (58.4%)	87 (49.5%)	1.29	0.25
Failure to execute the prescription	D1 to D4	79 (49.4%)	79 (50.3%)	0.01	1.00
	D5+	112 (54.1%)	84 (47.7%)	0.79	0.37
Failure to follow the vomiting protocol	D1 to D4	12 (7.5%)	8 (5.0%)	0.30	0.57
	D5+	38 (18.4%)	13 (7.4%)	5.93	0.01
Others	D1 to D4	1 (0.6%)	1 (0.7%)	0.01	1.00
	D5+	5 (2.4%)	2 (1.1%)	0.01	1.00
Indeterminate	D1 to D4	7 (4.4%)	7 (4.4%)	0.01	1.00
	D5+	14 (6.8%)	12 (6.8%)	0.01	1.00

*Total of 160 attendances in D1 to D4 and 207 in D5+.

*Total of 157 attendances in D1 to D4 and 176 in D5+. GIT – Gastrointestinal tract.

The evaluation of the descriptive results in Table 4 allows us to observe that, in both periods evaluated (Ppi and Pi), “fasting” and “failure to execute the prescription” were the factors that most interfered with care, both in the first 4 days of ENT as from D5, being the factors with the highest frequency of occurrence in Ppi and Pi. According to the McNemar test, the intervention was able to significantly reduce the frequency of occurrence of the factor “failure to follow the vomiting protocol” from D5, in Pi, signaling its positive impact on this factor.

## DISCUSSION

The majority of nursing professionals were present in the three training cycles, with their participation increasing throughout Pi, reaching a higher percentage in C3. The planning of training, with prior scheduling with the sector coordination departments, and carried out during working hours and at work stations, added to the incentive to multiply the content covered, possibly contributed to this adherence rate.

In a study^(18)^ in which civil servants from a university were interviewed, the professionals reported feeling the need to share what they learned during their learning trajectories and, also, seek to resolve any doubts about certain content in theoretical materials or through contact with professionals considered a reference on the theme. The author also observed that social interaction among the employees consisted of a way of acquiring relevant knowledge, since social interaction with colleagues allowed learning, including through the imitation of good practices.

In this study, it is believed that the request for content dissemination was met and, furthermore, that carrying out the training in 3 cycles allowed the participation of more employees and encouraged the participation of professionals in more than one training, providing opportunities for recycling/refreshing content and clarifying doubts. Encouraging the active participation of professionals in the collective analysis of their work process contributes to mutual accountability for the production of care. This proposal is consistent with Permanent Health Education (*EPS*), which encourages collective analysis of problems and difficulties related to social and work practices in the daily life of organizations^(19)^.

On most ENT days, in both Ppi and Pi, calorie and protein provision met the guidelines of the American and European Societies for Parenteral and Enteral Nutrition^(1,2)^, with a caloric-protein supply greater than 80% of the stipulated goals. Therefore, nutritional support was adequate throughout the period considered. Other research that evaluated the quality of ENT in hospital units highlighted the difficulty in providing adequate nutritional support, demonstrating caloric inadequacies of 67.4%^(8)^ and 60.5%^(20)^. These findings contrast with the results found in this research, highlighting the quality of care provided to patients on ENT admitted to the institution evaluated.

Despite the fact that nutritional support was already adequate before the start of the interventions, the results found show that carrying out the interventions was able to increase the quality of ENT, as it led to a significant reduction in the total number of non-conforming days from D5 onwards of ENT and, also, a significant reduction in the number of inadequate days (≤80%) both in D1 to D4 and in D5+. In a study evaluating the effectiveness of a multifaceted nutritional educational intervention, aimed at medical professionals, on the quality of ENT in an ICU, a significant improvement in nutritional adequacy was observed after the intervention, with an increase from 74.2% to 96.2%^(21)^, corroborating the results of the present study.

Reducing nutritional deficits can contribute to better clinical outcomes for patients undergoing ENT, such as: reduction in length of stay^(8)^, in the incidence of infections and in the mortality^(1,2)^ and the need for mechanical ventilation^(22)^. The development of nutritional protocols capable of contributing to reducing the occurrence of factors that interrupt nutritional support should be prioritized to reduce nutritional deficits^(11)^. In this research, in addition to the institutional vomiting protocol, the teams were guided on good practices related to the administration of ENT, including procedures for maintaining patency and positioning of catheters, satisfactory execution of dietary prescriptions, and diarrhea management.

The guidelines provided during the interventions were efficient in reducing the interference caused by the factors, with a significant reduction from D5 of ENT onwards, when these occurred more often. These findings corroborate the optimization of nutritional supply after training, demonstrated by the reduction in the number of non-conforming and inadequate days.

The most frequent factors that caused interruption of ENT and may have had an impact on caloric-protein supply were “fasting” and “failure to execute the prescription”, in Ppi and Pi; that is, there was no change in the main interruption factors after the intervention. In a longitudinal study^(23)^ in which the nutritional support provided to critically ill patients was evaluated for 5 years, no changes were observed in the main causes of ENT interruption.

Fasting has been identified as one of the main factors that interfere with nutritional supply, with percentages of occurrence varying between 90%^(4)^ and 51%^(24)^. A study carried out in an ICU that investigated the causes, duration, and frequency of ENT interruptions found that fasting for diagnostic procedures occurred in 100% of the sample and had a median duration of 3 hours^(20)^. Gastrointestinal symptoms^(4)^ and hemodynamic instability^(24)^ were also identified among the main causes for caloric-protein inadequacy, with occurrences of 10 and 20%, respectively. These data explain the variation in the literature regarding the contribution of different factors to nutritional supply and reinforce the result found in this research.

In some situations, the causes of interruption of enteral nutrition are avoidable as they are related to logistical problems and the interference of other professionals in the administration of ENT^(6)^. In this study, the “obstruction and externalization of the catheter”, “failure to follow the vomiting protocol”, “failure to execute the prescription” and the “indeterminate” factor (absence of registration) were the factors subject to modification by the training, as they are directly related to the care team behavior, having been extensively discussed in the 3 training cycles.

The “failure to execute the prescription” refers mainly to the discrepancy between the prescribed infusion volume and that used in the CIP, highlighting the non-compliance with the nutritional prescription caused by failure of the care team. It should also be emphasized that this factor most significantly affected the care provided in the first four days of ENT, when infusion volumes have to be updated in a daily basis.

Data classified as “indeterminate” points to an operational failure, as it represents a lack of record of the real reason for the ENT interruption, indicating the need to improve the documentation of information by the team. “Catheter obstruction”, in its turn, occurs when there is no adherence to the protocol for administering water to wash the catheter, as well as “catheter externalization”, resulting from the use of inadequate fixation^(10)^.

The use of targeted institutional protocols is seen as a measure capable of mitigating pauses in nutritional support caused by gastrointestinal symptoms^(25)^. In this research, the educational intervention was able to significantly reduce the frequency of interruption of nutritional support due to “failure to follow the vomiting protocol”, demonstrating that, despite the previous implementation of this document in the institution, there were still interferences in the supply of calories and proteins due to poor adherence. Detecting the occurrence of these factors reinforces the need for constant action by the multidisciplinary team in the search for strategies that minimize nutritional inadequacies.

A limitation of this study is that the sample was selected by convenience and not randomly, resulting in a profile of professionals that may be different from the profile found in other hospital units, which may compromise the generalization of the results. However, in general, this type of sampling is used due to the difficulty in accessing a random sample of the population of interest. The collection of data from medical records and instruments used by the institution’s professionals can also be considered a limitation, as there is the possibility of information bias, mainly due to incomplete records. The profile of the patients treated was not presented, but the dietary prescription is individualized and follows protocols, so this profile did not influence the analysis of the intervention effectiveness.

As a strength of the study, it is highlighted that it is a pioneer in the investigation of the effectiveness of training aimed at nursing professionals in relation to the interruption of the diet and its compliance and adequacy, as well as in the analysis of the frequency of occurrence of factors that lead to non-conformities in ENT administration. Our results reinforce the importance of these actions to improve the work of nursing teams in the administration of ENT.

## CONCLUSION

The training contributed to optimizing nutritional supply through ENT, with a significant reduction in days with inadequate caloric-protein supply and interference in ENT caused by the factors analyzed. Encouraging the active participation of professionals promoted greater involvement with teaching-­learning activities and was important for adherence to guidelines and best practices, allowing daily reflection on the care provided to patients. Therefore, dialogical educational interventions are a valid strategy to increase the effectiveness of ENT, contributing to better nutritional assistance.
