# Supplementary material for: Educational intervention with nursing professionals reduces interruption of enteral nutritional support
Source: Rev Esc Enferm USP. 2024 Sep 9;58:e20240132. doi: 10.1590/1980-220X-REEUSP-2024-0132en (PMC11391780; doi:10.1590/1980-220X-REEUSP-2024-0132en)
Supplement: Translation

# Translation: Intervenção educativa com profissionais de enfermagem reduz interrupção do suporte nutricional enteral*

**DOI:** 10.1590/1980-220X-REEUSP-2024-0132pt

**Authors:** Aline Oliveira Diniz, Igor Rosa Meurer, Kely Cristine Batista, Valesca Nunes dos Reis, Ana Paula Boroni Moreira, Silvia Lanziotti Azevedo da Silva

**Affiliations:** 1 Universidade Federal de Juiz de Fora, Hospital Universitário, Empresa Brasileira de Serviços Hospitalares, Juiz de Fora, MG, Brasil.; 2 Universidade Federal de Juiz de Fora, Hospital Universitário, Juiz de Fora, MG, Brasil.; 3 Universidade Federal de Juiz de Fora, Instituto de Ciências Biológicas, Departamento de Nutrição, Juiz de Fora, MG, Brasil.; 4 Universidade Federal de Juiz de Fora, Faculdade de Medicina, Departamento de Saúde Coletiva, Juiz de Fora, MG, Brasil.

**Keywords:** Nutrição Enteral, Ingestão de Energia, Intervenção Educacional Precoce, Enfermagem

## Abstract

**Objetivo:**

Avaliar o impacto de intervenção educativa na ocorrência de fatores que interferem na oferta calórica-proteica aos pacientes críticos e não críticos em terapia nutricional enteral.

**Método:**

Trata-se de uma intervenção, do tipo experimento de campo sem grupo controle, realizada em um hospital de ensino de Juiz de Fora, Minas Gerais, Brasil. Foram realizados 3 ciclos de capacitações com profissionais de enfermagem ao longo de 57 semanas, com abordagem dos mesmos conteúdos. Os dados coletados foram divididos em período pré-intervenção e de intervenção. A interferência no suporte calórico-proteico foi analisada a partir da avaliação dos dias com oferta nutricional não conforme e da ocorrência dos fatores que levaram às não conformidades.

**Resultados:**

Após as intervenções, houve redução significativa (p < 0,05) do número de dias não conformes e inadequados (oferta nutricional < 80%), da frequência de ocorrência dos fatores de interferência e das inconformidades ocasionadas pela não execução do protocolo de vômito.

**Conclusão:**

A intervenção educativa foi uma estratégia efetiva para aumentar a qualidade da terapêutica avaliada, especialmente a partir do quinto dia de terapia nutricional enteral.

## INTRODUÇÃO

A associação entre Terapia Nutricional Enteral (TNE) e evolução clínica do paciente já está muito bem estabelecida, visto que é uma estratégia terapêutica capaz de reduzir gravidade da doença, complicações infecciosas, tempo de permanência hospitalar e mortalidade^(1,2)^. Tais benefícios estão condicionados a sua implementação precoce (primeiras 48 horas), controle glicêmico meticuloso e fornecimento adequado de calorias, macro e micronutrientes, sendo a TNE a via de administração preferencial, sempre que viável^(2)^.

Apesar dos benefícios, comumente os pacientes hospitalizados não recebem o suporte nutricional prescrito e suficiente para suprir suas demandas^(3)^. Alguns estudos avaliaram a oferta da dieta enteral em diferentes perfis de pacientes e verificaram inadequações em relação às prescrições calóricas e proteicas: duas pesquisas conduzidas em unidades de terapia intensiva constataram que 30%^(4)^ e 25%^(5)^ dos pacientes não alcançaram a meta calórica-proteica; uma pesquisa realizada em um hospital oncológico^(6)^ verificou que apenas 50,2% do volume de dieta prescrito foi recebido; e em outro estudo realizado com pacientes críticos^(7)^ os autores encontraram que, apesar de 92% dos pacientes receberem mais de 80% do volume programado, apenas 43,6% receberam mais de 80% da meta proteica, sendo encontrada associação entre inadequação proteica e mortalidade.

A ocorrência de déficits nutricionais deve-se a diversas condições que interferem na oferta nutricional planejada, causando sua suspensão temporária e/ou permanente, como sintomas gastrointestinais (vômito, diarreia, drenagem gástrica elevada, distensão abdominal, constipação), exteriorização/obstrução do cateter de alimentação, jejum para procedimentos e exames e instabilidade hemodinâmica^(6,8,9)^. Ainda são citadas práticas inadequadas dos profissionais da equipe multidisciplinar, como prescrição inadequada, início tardio da TNE e pausas desnecessárias, além da recusa do tratamento pelo paciente, como fatores que levam à interrupção da dieta^(4,10)^.

O conhecimento dos fatores que prejudicam a administração energético-proteica satisfatória permite a adoção de medidas que visem minimizar interrupções da dieta e assegurar a administração adequada da TNE. São exemplos desses fatores o aperfeiçoamento de protocolos nutricionais, estabelecimento de medidas de controle para complicações gastrointestinais, avaliação da duração da interrupção para exames e procedimentos, capacitação das equipes para melhor aplicação dos protocolos e melhor registro de informações^(5,9,11)^.

A nutrição é um campo de atuação importante para a enfermagem desde o século XIX, quando a fundadora da enfermagem moderna já realizava ações em prol da nutrição adequada e de boa qualidade^(12)^. Os cuidados de enfermagem são vitais para o sucesso da TNE. Dentre eles, podem ser citados o monitoramento do paciente e das complicações potenciais bem como o manuseio apropriado de tais complicações, a manutenção da funcionalidade do cateter, a realização da higiene oral e nasal, a redução do risco de aspiração e o registro adequado dos volumes de soluções administrados^(13,14)^.

A literatura disponível evidencia a necessidade de revisão das práticas e constante capacitação dos enfermeiros nos serviços de saúde, principalmente daqueles envolvidos com o cuidado de pacientes em uso de TNE, com a ampliação do saber prático nessa temática^(12,15)^. Considerando que a ocorrência de diversos fatores dificulta o alcance das metas nutricionais e, ainda, as lacunas existentes na formação dos profissionais da equipe assistencial, fica evidente a necessidade de aumentar a efetividade da TNE para garantir sua qualidade e seus benefícios para a evolução do paciente.

Dessa forma, o presente estudo visa avaliar se uma intervenção educativa dialógica direcionada aos profissionais de enfermagem envolvidos na administração da TNE é capaz de minimizar a ocorrência de fatores que interferem na oferta nutricional enteral a pacientes críticos e não críticos internados em um hospital universitário.

## MÉTODO

### Desenho do Estudo

Estudo do tipo experimento de campo sem grupo controle.

### Local do Estudo

A pesquisa foi realizada no Hospital Universitário da Universidade Federal de Juiz de Fora, cidade de Minas Gerais, Brasil.

### População-alvo do Experimento

O experimento foi direcionado aos profissionais de enfermagem que atuam na assistência aos pacientes adultos e idosos internados no Hospital.

### Estratégia Educativa: Realização das Capacitações

O estudo foi conduzido entre maio de 2021 a agosto de 2023, sendo dividido em dois períodos: período pré-intervenção (Ppi) e período de intervenção (Pi). A Figura 1 ilustra o desenho e os períodos do estudo.

**Figura 1:** Fluxograma dos períodos pré-intervenção e de intervenção.

A intervenção educativa consistiu na realização de três ciclos de capacitações desenvolvidos ao longo do período de intervenção (Pi), com intervalos de 19 semanas entre eles.

Objetivando alcançar maior número de enfermeiros e técnicos de enfermagem, as capacitações foram previamente agendadas com os coordenadores de cada setor (enfermarias e Unidade de Terapia Intensiva - UTI), ocorreram nos três turnos de trabalho (manhã, tarde e noite), em dias estratégicos, durante as jornadas e nos respectivos postos de trabalho dos funcionários, com tempo médio de duração de 30 minutos. Foi realizada uma capacitação para cada uma das 17 equipes de enfermagem participantes do estudo em cada um dos 3 ciclos, totalizando 51 capacitações ao final do estudo.

Tendo em vista que os profissionais de enfermagem trabalhavam em turnos diferentes (matutino e/ou vespertino ou noturno) e que alguns possivelmente estavam em usufruto de férias, folgas ou afastamento durante algum dos momentos de intervenção, os participantes capacitados foram encorajados a serem multiplicadores dos conteúdos abordados.

As capacitações foram ministradas pelas duas enfermeiras da Equipe Multiprofissional de Terapia Nutricional (EMTN) com suporte de uma nutricionista da referida equipe, utilizando a estratégia de Rodas de Conversas. Sendo assim, foram conduzidas de modo que consistissem em espaços de encontro entre trabalhadores das diferentes categorias profissionais com oportunidade para o diálogo e a reflexão sobre os fatores que implicam na administração inadequada da TNE, buscando a melhor conduta frente a eles^(16)^.

Os conteúdos base abordados nas capacitações foram elaborados segundo as Diretrizes de Enfermagem em Terapia Nutricional da Sociedade Brasileira de Nutrição Parenteral e Enteral (BRASPEN)^(14)^ e a Resolução de Diretoria Colegiada - RDC nº 503^(13)^, que dispõe sobre os requisitos mínimos para a TNE, sendo constituídos pelos seguintes tópicos: a) Manutenção da perviedade, técnicas de desobstrução e controle de posicionamento dos cateteres para TNE; b) Execução do protocolo institucional de vômito; c) Ocorrência de diarreia; d) Registro de informações; e) Manejo da bomba de infusão controlada (BIC); f) Instruções quanto à execução da prescrição nutricional.

Os mesmos conteúdos foram abordados nos três ciclos de capacitações, favorecendo a melhor fixação do conhecimento e padronização da metodologia empregada.

### Coleta de Dados

Os pacientes em TNE internados na instituição estudada são acompanhados diariamente pela EMTN. Os cálculos das necessidades calórica e proteica são realizados conforme os protocolos institucionais e seguem as recomendações das Diretrizes de TN de instituições como American Society for Parenteral and Enteral Nutrition (ASPEN)^(1)^, ESPEN (European Society for Clinical Nutrition and Metabolism)^(2)^ e Braspen (Brazilian Society of Parenteral and Enteral Nutrition)^(17)^.

A avaliação da efetividade desta intervenção sobre a administração da TNE se deu por meio da análise de variáveis relacionadas aos dias de interrupção da dieta com ou sem inadequação da oferta de calorias e proteínas e a ocorrência de fatores que interferem nesta oferta, sendo comparadas as mesmas variáveis nos dois períodos considerados. Desta forma, foram incluídas informações referentes a prescrição e infusão da TNE e fatores que interferiram na oferta da TNE a pacientes adultos e idosos (19 anos ou mais) de ambos os sexos, internados na UTI e nas enfermarias do hospital e que estiveram em uso de TNE via cateter exclusiva. Foram excluídas informações de pacientes que estiveram em TNE por menos de 72 horas, em gestação, em cuidados paliativos/doença terminal.

Os dados do Ppi foram coletados antes do início da primeira capacitação, pelo mesmo tempo do Pi (57 semanas). Para avaliar o impacto da intervenção na oferta nutricional, os dias de TNE foram avaliados de acordo com a conformidade ou não da oferta calórica-proteica com os valores prescritos, sendo considerados como dias não conformes aqueles em que houve qualquer interferência no suporte nutricional, levando a uma oferta calórica-proteica diferente da prescrita. Da mesma forma, a partir da avaliação dos dias não conformes, foram quantificados os dias em que a oferta calórica-proteica foi igual ou inferior a 80% do prescrito, visto que já foi demonstrado que a carga calórica ideal associada à melhor sobrevida é de cerca de 80% das necessidades energéticas previstas^(1,2)^. Foi também analisada a ocorrência ou não de fatores que interferiram na oferta da TNE em cada atendimento prestado pelas equipes: a critério médico, complicações relacionadas ao trato gastrointestinal (TGI), obstrução do cateter, exteriorização do cateter, intercorrência clínica, jejum, não execução da prescrição, não execução do protocolo de vômito, outros e indeterminado.

A fim de alcançar os benefícios clínicos da TNE durante a primeira semana de hospitalização, devem ser fornecidas mais de 80% das metas calóricas e proteicas estimadas em 48 a 72 horas após início do suporte nutricional e o aporte nutricional pleno deve ser prescrito de 3 a 7 dias a partir do início da TN^(1,2)^. Sendo assim, a progressão da velocidade de infusão das dietas enterais segue o protocolo estabelecido pela EMTN, iniciando-se com 50% (primeiro dia de TNE – D1) e evoluindo a cada 24 horas, após avaliação da equipe, para 65%, 80% e 100% das necessidades calculadas. Assim, a prescrição dietética alcança adequação de 100% às necessidades calóricas e proteicas no quinto dia (D5) de suporte nutricional. Dessa forma, buscando melhor elucidar em quais dias de TNE o aporte ofertado aos pacientes é mais afetado (ponto crítico), os atendimentos foram subdivididos de acordo com os dias de TNE: atendimentos realizados do primeiro (D1) ao quarto dia (D4) de TNE e atendimentos realizados a partir do quinto dia (D5).

Os dados coletados são referentes aos atendimentos prestados pelas equipes de Nutrição e Enfermagem, cujo padrão é independente do perfil do paciente ao qual são direcionados uma vez que são determinados pelos protocolos institucionais.

### Análise Estatística

Foram calculados valores de média e desvio padrão do número de dias não-conformes, especificando aqueles dias com oferta adequada (>80%) e inadequada (≤80%), e do número de ocorrência dos fatores que interferiram na oferta da TNE em Ppi e Pi. Também foram calculadas as frequências absoluta e relativa de ocorrência de cada um destes fatores nos atendimentos prestados pelas equipes nos dois períodos de coleta.

Para avaliação da efetividade da intervenção, as mesmas variáveis foram consideradas em Ppi e Pi. A associação entre os dois períodos de avaliação e a possível efetividade da intervenção foi verificada utilizando-se modelos de regressão lineares mistos. De acordo com os valores do coeficiente Beta em Pi, comparado ao Ppi, foi possível identificar se houve aumento ou diminuição média de cada variável no Pi, tanto nos primeiros quatro dias de TNE (D1 a D4) quanto a partir do quinto dia (D5+). Os modelos lineares mistos permitem analisar o efeito fixo da intervenção realizada, controlando pelo efeito aleatório decorrente do perfil dos pacientes atendidos em cada período. O controle por efeito aleatório no modelo linear misto minimiza eventuais efeitos das características individuais dos pacientes, que não eram os mesmos em todos os períodos da intervenção. O efeito fixo, aquele que é realmente avaliado, é o da efetividade da intervenção educativa sobre o atendimento prestado, este sim, realizado pelos mesmos profissionais, população-alvo da intervenção, em todos os períodos.

Para comparação das frequências de interrupção por cada um dos fatores estudados entre o Ppi e Pi foi utilizado o teste de McNemar para comparação de proporções. O teste foi escolhido em função da dependência entre os momentos considerando-se que as intervenções eram aplicadas pelas mesmas equipes.

Para a interpretação estatística dos resultados, foi considerado o intervalo de confiança de 95% (IC95%) e em todos os testes foi adotado nível de significância alfa inferior a 0,05. As análises foram realizadas no pacote estatístico R 4.3.1.

### Aspectos Éticos

O Comitê de Ética em Pesquisa com Seres Humanos do Hospital Universitário da Universidade Federal de Juiz de Fora aprovou a pesquisa mediante parecer técnico número 4.825.877. Os profissionais que aceitaram participar deste estudo forneceram seu consentimento por meio da assinatura do Termo de Consentimento Livre e Esclarecido (TCLE).

## RESULTADOS

Foram realizadas 51 capacitações no decorrer do período de intervenção (Pi), que contaram com a participação de 58 enfermeiros e 202 técnicos de enfermagem, totalizando 260 profissionais capacitados.

Houve um aumento gradativo da participação dos profissionais ao longo dos três ciclos de capacitação (C1, C2 e C3): 55,33% dos profissionais Enfermeiros e Técnicos de Enfermagem participaram de C1, 61,48% participaram de C2 e o maior alcance ocorreu em C3, quando participaram 64,75% dos profissionais.

Entre os 58 enfermeiros que participaram das capacitações: 18 (31%) participaram de uma capacitação, 22 (38%) de duas capacitações e 18 (31%) de três capacitações. Quanto aos 202 técnicos de enfermagem: 106 (52,5%), 67 (33,2%) e 29 (14,3%) participaram, respectivamente, de uma, duas ou três capacitações.

Considerando que as capacitações também tiveram a proposta de favorecer a comunicação e a integração entre as equipes de Nutrição e Enfermagem assistencial, em algumas ocasiões, a depender da participação dos sujeitos, o tempo de duração das capacitações ultrapassou o previsto, pois algumas equipes interagiram mais que outras.

No Ppi foram avaliados 367 atendimentos, sendo 160 atendimentos de D1 a D4 e 207 em D5+; e no Pi foram avaliados 333 atendimentos, 157 de D1 a D4 e 176 em D5+.

Na Tabela 1, os atendimentos são avaliados quanto ao número de dias não conformes com oferta calórica-proteica inadequada (≤80% das metas prescritas) e adequada (>80% das metas prescritas).

**Tabela 1:** Média (±DP) dos dias não conformes nos atendimentos avaliados nos períodos pré-intervenção (Ppi) e de intervenção (Pi) – Juiz de Fora, MG, Brasil, 2022-2023.

Variável	Dias de TNE	Ppi			Pi		
		n	%*	Média ± DP	n	%**	Média ± DP
Total de dias não conformes	D1 a D4 e D5+	1220	29,93	5,35 ± 4,50	895	29,12	4,48 ± 4,14
	D1 a D4	255	39,84	1,59 ± 15,81	239	38,06	1,52 ± 1,10
	D5+	965	28,08	4,66 ± 0,95	656	26,82	3,73 ± 4,06
Dias não conformes e inadequados (≤80%)	D1 a D4	114	17,81	0,71 ± 1,19	81	12,90	0,52 ± 0,71
	D5+	533	15,51	2,57 ± 4,40	354	14,47	2,01 ± 2,51
Dias não conformes e adequados (>80%)	D1 a D4	141	22,03	0,88 ± 0,90	158	25,16	1,01 ± 0,96
	D5+	432	12,57	2,09 ± 2,57	302	12,35	1,72 ± 2,10

*Em relação aos 4076 dias avaliados, sendo 640 dias de D1 a D4 e 3436 em D5+.

**Em relação aos 3074 dias avaliados, sendo 628 dias de D1 a D4 e 2446 em D5+. TNE – Terapia Nutricional Enteral, Ppi – Período pré-intervenção, Pi – Período de intervenção.

É possível afirmar que houve conformidade da oferta de calorias e proteínas com a prescrição dietética na maioria dos dias de TNE nos dois períodos avaliados: em 2856 (70,07%) e 2179 (70,88%) dias avaliados em Ppi e Pi, respectivamente, a oferta calórica-proteica foi semelhante aos valores prescritos. Por sua vez, a inadequação calórica-proteica (oferta ≤80% das metas) ocorreu em 647 dias (15,87%) no Ppi e 435 dias (14,15%) no Pi. Nota-se, ainda, que no Pi houve redução dos valores das médias para todas as variáveis analisadas, exceto para os dias não conformes e adequados avaliados nos primeiros 4 dias de TNE (Tabela 1).

Na Tabela 2, são apresentadas as médias do número de fatores, considerando todos os estudados em conjunto, que interferiram na oferta da TNE nos atendimentos avaliados. É possível constatar que os fatores interferiram mais nos atendimentos prestados a partir do quinto dia de TNE e, ainda, que houve queda das médias do número de fatores no Pi.

**Tabela 2:** Média (±DP) da frequência de ocorrência dos fatores que interferiram na oferta da TNE nos atendimentos avaliados nos períodos pré-intervenção (Ppi) e de intervenção (Pi) – Juiz de Fora, MG, Brasil, 2022-2023.

Variável	Dias de TNE	Ppi		Pi	
		n	Média ± DP	n	Média ± DP
Número de fatores de interferência	D1 a D4	307	1,92 ± 2,56	286	1,82 ± 1,49
	D5+	1108	5,33 ± 5,39	727	4,13 ± 4,77

TNE – Terapia Nutricional Enteral, Ppi – Período pré-intervenção, Pi – Período de intervenção.

Conforme demonstrado na Tabela 3, os valores de Beta evidenciaram redução da média do número de dias não conformes e inadequados no Pi, tanto nos primeiros dias de TNE (D1 a D4) quanto nos dias subsequentes (D5+), com diferença estatisticamente significativa. Ainda, os valores de Beta demonstraram redução da média do número de fatores que interferiram na oferta nutricional a partir do D5 de TNE no Pi, com significância estatística (p = 0,02).

**Tabela 3:** The average percentage of wound healing of rats with pressure injury – Gansu, China, 2020.

Variável	Dia de TNE	Efeito aleatório individual	Efeito fixo			
			Intercepto	Beta 1	S.E.	p valor
Total de dias não conformes	D1 a D4 e D5+	4,85	17,68	–2,63	1,38	0,06
	D1 a D4	0,00	0,00	0,00	0,00	1
	D5+	5,12	16,31	–4,40	1,41	0,00
Dias não conformes e inadequados (=80%)	D1 a D4	0,05	0,71	–0,19	0,09	0,03
	D5+	0,75	2,55	–0,55	0,25	0,02
Dias não conformes e adequados (>80%)	D1 a D4	0,12	0,88	0,12	0,10	0,24
	D5+	0,80	2,04	–0,36	0,22	0,11
Número de fatores de interferência	D1 a D4	0,00	1,91	–0,09	0,18	0,59
	D5+	1,55	5,27	–1,16	0,50	0,02

TNE – Terapia Nutricional Enteral, S.E. – Standard error.

A Tabela 4 descreve as frequências absolutas e relativas de cada fator de interrupção nos atendimentos, considerando o total de atendimentos avaliados em cada período. Convém destacar que um mesmo atendimento pode ser acometido, simultaneamente ou não, por mais de um fator de interferência. A tabela também apresenta a comparação dessas frequências entre o Ppi e Pi pelo teste McNamar.

**Tabela 4:** Ocorrência dos fatores que interferiram na TNE nos atendimentos prestados nos períodos pré-intervenção (Ppi) e de intervenção (Pi) – Juiz de Fora, MG, Brasil, 2022-2023.

Fatores de interferência	Dias de TNE	Atendimentos que sofreram interferência		McNamar	p valor
		Ppi*	Pi**		
A critério médico	D1 a D4	15 (9,4%)	9 (5,7%)	0,01	1,00
	D5+	33 (15,9%)	25 (14,2%)	0,25	0,61
Complicações do TGI	D1 a D4	14 (8,8%)	15 (9,5%)	0,01	1,00
	D5+	65 (31,4%)	42 (23,9%)	1,04	0,30
Obstrução do cateter	D1 a D4	5 (3,1%)	3 (1,9%)	0,8	0,37
	D5+	33 (15,9%)	14 (7,9%)	3,55	0,06
Exteriorização do cateter	D1 a D4	21 (13,1%)	21 (13,4%)	0,01	1,00
	D5+	63 (30,4%)	55 (31,2%)	0,40	0,52
Intercorrência clínica	D1 a D4	8 (5,0%)	10 (6,4%)	0,01	1,00
	D5+	51 (24,6%)	31 (17,6%)	1,76	0,18
Jejum	D1 a D4	38 (23,7%)	37 (23,6%)	0,37	0,54
	D5+	121 (58,4%)	87 (49,5%)	1,29	0,25
Não execução da prescrição	D1 a D4	79 (49,4%)	79 (50,3%)	0,01	1,00
	D5+	112 (54,1%)	84 (47,7%)	0,79	0,37
Não execução do protocolo de vômito	D1 a D4	12 (7,5%)	8 (5,0%)	0,30	0,57
	D5+	38 (18,4%)	13 (7,4%)	5,93	0,01
Outros	D1 a D4	1 (0,6%)	1 (0,7%)	0,01	1,00
	D5+	5 (2,4%)	2 (1,1%)	0,01	1,00
Indeterminado	D1 a D4	7 (4,4%)	7 (4,4%)	0,01	1,00
	D5+	14 (6,8%)	12 (6,8%)	0,01	1,00

*Total de 160 atendimentos em D1 a D4 e 207 em D5+.

**Total de 157 atendimentos em D1 a D4 e 176 em D5+. TGI – Trato gastrointestinal.

A avaliação dos resultados descritivos da Tabela 4 permite constatar que, em ambos os períodos avaliados (Ppi e Pi), o “jejum” e a “não execução da prescrição” foram os fatores que mais interferiam nos atendimentos, tanto nos primeiros 4 dias de TNE quanto a partir de D5, sendo os fatores com maiores frequências de ocorrência em Ppi e Pi. De acordo com o teste de McNemar, a intervenção foi capaz de reduzir significativamente a frequência de ocorrência do fator “não execução do protocolo de vômito” a partir de D5, no Pi, sinalizando para seu impacto positivo neste fator.

## DISCUSSÃO

Nos três ciclos de capacitação esteve presente a maioria dos profissionais de enfermagem, tendo havido um aumento crescente da participação destes no decorrer do Pi, alcançando maior percentual em C3. O planejamento das capacitações, com agendamento prévio junto às coordenações dos setores e realização durante as jornadas e nos postos de trabalho, somado ao incentivo à multiplicação dos conteúdos abordados possivelmente contribuíram para esta taxa de adesão.

Em um estudo^(18)^ em que os funcionários públicos de uma universidade foram entrevistados, os profissionais relataram sentir a necessidade de compartilhar o que aprenderam durante suas trajetórias de aprendizado e, ainda, buscar sanar eventuais dúvidas sobre determinado conteúdo em materiais teóricos ou por meio do contato com profissionais considerados referência na temática. O autor também observou que o convívio entre os funcionários consistiu numa forma de aquisição de conhecimento relevante, uma vez que a interação social com os colegas possibilitou o aprendizado, inclusive por meio da imitação de boas práticas.

Neste estudo, acredita-se que a solicitação de divulgação dos conteúdos foi atendida e, ainda, que a realização das capacitações em 3 ciclos possibilitou a participação de mais funcionários e incentivou a participação dos profissionais em mais de uma capacitação, oportunizando a reciclagem/revisão dos conteúdos e o esclarecimento de dúvidas. O encorajamento à participação ativa dos profissionais na análise coletiva do seu processo de trabalho contribui para a responsabilização mútua pela produção de cuidados. Esta proposta condiz com a Educação Permanente em Saúde (EPS), que encoraja a análise coletiva dos problemas e dificuldades relacionados às práticas sociais e laborais na vida cotidiana das organizações^(19)^.

Na maioria dos dias de TNE, tanto no Ppi quanto no Pi, o fornecimento de calorias e proteínas atendeu às diretrizes das sociedades americana e europeia de nutrição parenteral e enteral^(1,2)^, com oferta calórica-proteica superior a 80% das metas estipuladas. Portanto, o suporte nutricional foi adequado durante todo o período considerado. Outras pesquisas que avaliaram a qualidade da TNE em unidades hospitalares evidenciaram a dificuldade em fornecer um suporte nutricional adequado, demonstrando inadequações calóricas de 67,4%^(8)^ e 60,5%^(20)^. Estes achados contrapõem-se aos resultados encontrados nesta pesquisa, ressaltando a qualidade dos atendimentos prestados aos pacientes em TNE internados na instituição avaliada.

Apesar do fato de que o suporte nutricional já estava adequado antes do início das intervenções, os resultados encontrados mostram que a realização das intervenções foi capaz de aumentar a qualidade da TNE, pois levou a redução significativa do total de dias não conformes a partir do D5 de TNE e, ainda, redução significativa do número de dias inadequados (≤80%) tanto em D1 a D4 quanto em D5+. Em um estudo que avaliou a efetividade de uma intervenção educativa nutricional multifacetada, direcionada aos profissionais médicos, na qualidade da TNE em uma UTI, foi observada melhora significativa da adequação nutricional após a intervenção, com aumento de 74,2% para 96,2%^(21)^, corroborando os resultados do presente estudo.

A redução dos déficits nutricionais pode contribuir para melhores desfechos clínicos de pacientes em TNE, como: redução do tempo de internação^(8)^, da incidência de infecções e mortalidade^(1,2)^ e da necessidade de ventilação mecânica^(22)^. O desenvolvimento de protocolos nutricionais capazes de contribuir para redução da ocorrência de fatores que interrompem o suporte nutricional deve ser priorizado a fim de reduzir os déficits nutricionais^(11)^. Nesta pesquisa, além do protocolo institucional de vômito, as equipes foram orientadas quanto às boas práticas relacionadas a administração da TNE, incluindo procedimentos para manutenção da perviedade e posicionamento dos cateteres, execução satisfatória da prescrição dietética e manejo da diarreia.

As orientações realizadas durante as intervenções foram eficientes para reduzir as interferências ocasionadas pelos fatores, tendo havido redução significativa a partir do D5 de TNE, quando estes ocorreram de forma mais expressiva. Estes achados corroboram a otimização da oferta nutricional após realização das capacitações, demonstrada pela redução do número de dias não conformes e inadequados.

Os fatores mais frequentes que geraram interrupção da TNE e podem ter gerado impacto na oferta calórica-proteica foram o “jejum” e a “não execução da prescrição”, no Ppi e no Pi; ou seja, não houve modificação dos principais fatores de interrupção após a intervenção. Em um estudo longitudinal^(23)^ em que o suporte nutricional ofertado aos pacientes críticos foi avaliado durante 5 anos, também não foi observada alteração das principais causas de interrupção da TNE.

O jejum tem sido apontado como um dos principais fatores que interferem na oferta nutricional, com percentuais de ocorrência variando entre 90%^(4)^ e 51%^(24)^. Estudo realizado em uma UTI que investigou as causas, a duração e a frequência das interrupções da TNE detectou que o jejum para procedimentos diagnósticos ocorreu em 100% da amostra e teve duração mediana de 3 horas^(20)^. Os sintomas gastrointestinais^(4)^ e a instabilidade hemodinâmica^(24)^ também foram identificados dentre as principais causas para inadequação calórica-proteica, com ocorrências de 10 e 20%, respectivamente. Estes dados explicitam a variação existente na literatura quanto à contribuição de diversos fatores na oferta nutricional e reforçam o resultado encontrado nesta pesquisa.

Em algumas situações, as causas de interrupção da nutrição enteral são evitáveis pois se relacionam com problemas logísticos e a interferência de outros profissionais na administração da TNE^(6)^. Nesta pesquisa, a “obstrução e exteriorização do cateter”, a “não execução do protocolo de vômito”, a “não execução da prescrição” e o fator “indeterminado” (ausência de registro) foram os fatores passíveis de modificação pelas capacitações, pois têm relação direta com as condutas da equipe assistencial, tendo sido extensamente discutidos nos 3 ciclos de capacitações.

A “não execução da prescrição” refere-se, principalmente, à discrepância entre o volume de infusão prescrito e o utilizado na BIC, evidenciando a inconformidade com a prescrição nutricional ocasionada por falha da equipe assistencial. Enfatiza-se, ainda, que este fator afetou mais expressivamente os atendimentos prestados nos primeiros quatro dias de TNE, quando há necessidade de atualização diária dos volumes de infusão.

Os dados classificados como “indeterminado” apontam para falha operacional, pois representam ausência de registro do real motivo da interrupção da TNE, indicando a necessidade de melhorar a documentação das informações por parte da equipe. A “obstrução do cateter”, por sua vez, ocorre quando não há adesão ao protocolo de administração de água para lavagem do cateter; bem como a “exteriorização do cateter”, decorrente do uso de fixação inadequada^(10)^.

A utilização de protocolos institucionais direcionados é apontada como medida capaz de mitigar as pausas no suporte nutricional ocasionadas pelos sintomas gastrointestinais^(25)^. Nesta pesquisa, a intervenção educativa foi capaz de reduzir significativamente a frequência de interrupção do suporte nutricional por “não execução do protocolo de vômito”, demonstrando que, apesar da implementação prévia deste documento na instituição, ainda ocorriam interferências na oferta de calorias e proteínas devido a adesão deficiente. A detecção da ocorrência destes fatores reforça a necessidade de atuação constante da equipe multiprofissional na busca por estratégias que minimizem as inadequações nutricionais.

Pode ser citada como limitação deste estudo o fato de a amostra ser selecionada por conveniência e não de forma aleatória, resultando num perfil de profissionais que pode ser diferente do perfil encontrado em outras unidades hospitalares, o que pode comprometer a generalização dos resultados. Porém, em geral, recorre-se a esse tipo de amostragem devido à dificuldade em acessar uma amostra aleatória da população de interesse. A coleta de dados de prontuários e instrumentos utilizados pelos profissionais da instituição também pode ser considerada uma limitação, pois existe a possibilidade de ocorrência de viés de informação, principalmente por registro incompleto. O perfil dos pacientes atendidos não foi apresentado, mas a prescrição dietética é individualizada e segue protocolos, logo tal perfil não influenciou na análise da efetividade da intervenção.

Como ponto forte destaca-se que este estudo é pioneiro na investigação da efetividade de capacitações direcionadas aos profissionais de enfermagem em relação à interrupção da dieta e sua conformidade e adequação, além da análise da frequência de ocorrência de fatores que levam a inconformidades na administração da TNE. Nossos resultados reforçam a importância dessas ações para melhoria do trabalho das equipes de enfermagem na administração da TNE.

## CONCLUSÃO

As capacitações contribuíram para otimizar a oferta nutricional por meio da TNE, havendo redução significativa dos dias com oferta calórica-proteica inadequada e das interferências na TNE ocasionadas pelos fatores analisados. O estímulo à participação ativa dos profissionais promoveu maior envolvimento com as atividades de ensino-aprendizagem e foi importante para a adesão às orientações e às melhores práticas, possibilitando a reflexão diária da assistência prestada aos pacientes. Sendo assim, as intervenções educativas dialógicas são uma estratégia válida para aumentar a efetividade da TNE, contribuindo para uma melhor assistência nutricional.
